# Detection of (In)activity Periods in Human Body Motion Using Inertial Sensors: A Comparative Study

**DOI:** 10.3390/s120505791

**Published:** 2012-05-04

**Authors:** Alberto Olivares, Javier Ramírez, Juan M. Górriz, Gonzalo Olivares, Miguel Damas

**Affiliations:** 1 Department of Signal Theory, Networking and Communications, University of Granada, ETSIIT, C/Periodista Daniel Saucedo Aranda s/n, E-18071, Granada, Spain; E-Mails: javierrp@ugr.es (J.R.); gorriz@ugr.es (J.M.G.); 2 Department of Computer Architecture and Computer Technology, University of Granada, ETSIIT, C/Periodista Daniel Saucedo Aranda s/n, E-18071, Granada, Spain; E-Mails: gonzalo@ugr.es (G.O.); mdamas@atc.ugr.es (M.D.)

**Keywords:** activity detection, inertial sensors, human body monitoring, activity recognition, IMU, ZUPT, calibration

## Abstract

Determination of (in)activity periods when monitoring human body motion is a mandatory preprocessing step in all human inertial navigation and position analysis applications. Distinction of (in)activity needs to be established in order to allow the system to recompute the calibration parameters of the inertial sensors as well as the Zero Velocity Updates (ZUPT) of inertial navigation. The periodical recomputation of these parameters allows the application to maintain a constant degree of precision. This work presents a comparative study among different well known inertial magnitude-based detectors and proposes a new approach by applying spectrum-based detectors and memory-based detectors. A robust statistical comparison is carried out by the use of an accelerometer and angular rate signal synthesizer that mimics the output of accelerometers and gyroscopes when subjects are performing basic activities of daily life. Theoretical results are verified by testing the algorithms over signals gathered using an Inertial Measurement Unit (IMU). Detection accuracy rates of up to 97% are achieved.

## 1. Introduction

Large amounts of works related with Ubiquitous Computing and Ambient Intelligence (AmI) are appearing in the literature within the past years [1] where we can see that there is an actual trend in applying technology to monitor and analyze many different aspects of human daily life. Studying how humans move and interact with their environment is an important part of Pervasive Health, AmI and Ubiquitous applications, like the telerehabilitation [2]. Thus, many efforts are being put to analyze human motion using different means (inertial sensors [3,4], camera-surveilled environments [5], a combination of both vision and inertial sensing [6], or robots following persons [7]). Monitoring human motion using cameras has shown to be very effective in representing motion characteristics, but presents issues with privacy and limitation of its application to closed spaces. Privacy is very important when developing Ubiquitous and Healthcare applications [8]. Therefore, many researchers have opted to develop human body motion monitoring systems based on inertial sensors since the subjects under study do not feel observed.

Detection of human body movement and inactivity periods is a critical step for human body monitoring applications. When body movement is being monitored using inertial or MARG (Magnetic, Angular Rate and Gravity) sensors, their output signals can be used to discriminate periods where the subject being monitored is static from those where he is moving. This distinction is imperative for sensor calibration and different motion monitoring applications like inertial navigation and human activity classifiers.

**Figure 1.**
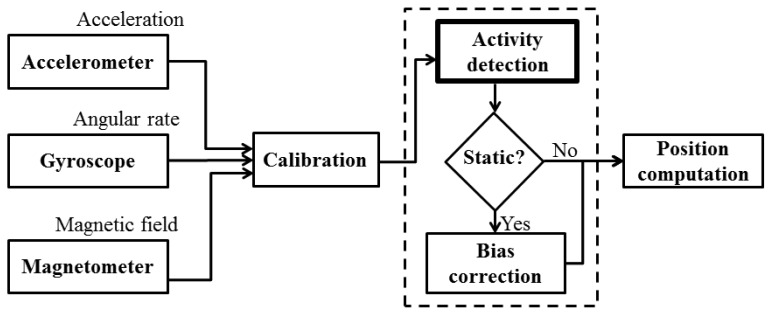
General diagram of positioning angles computation system based on inertial sensors. (In)activity detection is applied before position computation to allow correction of drifting parameters.

Most sensors present random time variations in the parameters of their mathematical model, such as the scale factors or biases [9,10]. Some works show different techniques to reduce drifts in inertial measurements using Kalman filtering [11] as well as other adaptive filtering algorithms [12]. Such a drifting behavior requires the periodical recomputation of the model parameters in order to maintain a satisfactory degree of precision during the complete monitoring session [13]. However, we can only recalculate them when there is neither acceleration nor angular velocity, for example, when the subject that is carrying them is stationary, since we need to know the zero level noise signal. Figure 1 shows the general diagram of systems based on inertial sensors used to compute positioning angles (pitch and roll). The determination of absolute positions also needs altitude estimates in addition to a digital compass to compute the yaw angle. Notice how the (in)activity detection needs to be applied prior to the computation of the angles describing the body position.

Inertial navigation applications also need to reset the offset parameters and perform corrections during static periods in order to help avoid erroneous drift in the trajectory of the subject [14–16].

Detecting static periods is, thus, a mandatory step in most inertial sensors applications.

Detection algorithms can be classified according to the sensor they use as an input. The Acceleration Moving Variance Detector (AMVD) proposed in [17] and the Acceleration Magnitude Detector (AMD) implemented in [18] use the acceleration signals to carry out the classification. This fact may limit the detection of possible instants where there is no acceleration but the gyros are measuring angular rate. On the other hand, the Angular Rate Energy Detector (ARED) employed in [19] uses the angular velocity signals as the input, which may also lead to erroneous classification of moments where there is little or no angular rate but accelerometers are sensing acceleration, as in inactivity periods. The Stance Hypothesis Optimal Detector (SHOD) proposed in [20] uses both the acceleration and angular velocity signals to increase the precision of the detector and, finally, the Filtered Rectifier Detector (FRD) employed in [17] has a flexible input (acceleration and angular rate magnitudes or a linear combination of both).

A comparative study among some of the aforementioned algorithms is also presented in [20], where a performance comparison of the detectors using real signals gathered from different sensors is shown. The mathematical definition of the detectors is very rigorous, however, as the authors state, the amount of signals used to compare the methods is rather low, making the study non-optimal in statistical terms.

The goal of the present work is to complete the comparative study among the previously mentioned methods over a large range of signals, in order to ensure statistical robustness. Due to the infeasibility of obtaining many signals gathered from different subjects performing a set of predetermined activities and hand labeling the start and end points of each activity/inactivity period, we have developed an acceleration and angular velocity signal synthesizer. This synthesizer will allow us to perform Monte Carlo tests over a large number of signals, making the study statistically representative.

In addition to using a larger data set, we have also completed the comparative study by implementing and testing four more detection methods. The first two are based on the computation of the spectrum (Fourier transform) of the input signals. We will use the Long Term Spectral Detector (LTSD) presented in [21] and a variation that we will refer as to the Framed Spectrum Detector (FSD). Spectrum-based methods have been widely used with success in Voice Activity Detection (VAD) applications [22–24]. By applying such algorithms we aim to find other possible detectors that may outperform those in [20], as they are very robust in conditions of low SNR. The last two methods that we will test are thought to detect abrupt changes in signals coming from sensors located in an industrial environment. These are the Memory-Based Graph Theoretic Detector (MBGTD) and the Memory-Based Cumulative Sum Detector (MBCD), both developed in [25]. Therefore, this work also presents the first results of the application of LTSD, FSD, MBGTD and MBCD algorithms in the detection of (in)activity periods of human body using inertial sensors.

This paper is organized as follows. Section 2 briefly presents the different detection methods that will be tested in the comparative study. Section 3 shows both the simulations and the application of the algorithms on real signals. Section 4 analyzes both results from theoretical and real experiments and compares our results to with those obtained in previous studies. Section 5 draws the conclusions and future evolution of the research.

## 2. (In)activity Detection Methods

As said in the introduction, we will be testing nine different methods. These methods can be grouped in three different sets: those based on the magnitude of the acceleration and/or the angular rate (AMVD, AMD, ARED, SHOD and FRD); those based on the spectrum of the acceleration and the angular rate (LTSD and FSD); those based on abrupt changes in data distributions (MBGTD and MBCD). The following subsections present the mathematical core of each of the detectors that is essential to program them, *i.e.*, we derive the expressions of the figures of merit that are used for the classification. At the end of the section, we have also included the workflow of the algorithms explaining step by step their general structure to ease their understanding.

### 2.1. Magnitude-Based Methods

The following methods use the magnitude of the acceleration, the magnitude of the angular velocity or a linear combination of both as the input signal. All the computations are carried out in the time domain of the signals.

#### 2.1.1. Acceleration Moving Variance Detector (AMVD)

The AMVD exclusively uses the acceleration signals to carry out the distinction of (in)activity periods. A sliding window is applied over the signal in which the variance of the acceleration is computed. The figure of merit of the detection algorithm is computed as follows,

(1) $$V(\text{n})=\frac{1}{N}\sum_{k=1}^{N}{\left\|{\text{a}}_{k}-{\bar{\text{a}}}_{n}\right\|}^{2}<\gamma$$

where **n** is the frame at instant *n, i.e.*, the content of the sliding window at instant *n*, **a***_k_* is the acceleration vector at instant *k*, **a(x00304)***_n_* is the mean of the acceleration of the frame at instant *n, N* is the length of the frame and *γ* is the predefined threshold that characterizes the decision based on the resultant value of the figure of merit.

#### 2.1.2. Acceleration Magnitude Detector (AMD)

The AMD is also solely based on the acceleration signals. The magnitude of the gravity acceleration vector is subtracted from the magnitude of the acceleration vector which is computed at every instant. The figure of merit used as the input of the classifier can be computed as

(2) $$V(\text{n})=\frac{1}{\sigma_{\text{a}}^{2}N}\sum_{k=1}^{N}{\left(\left\|{\text{a}}_{k}\right\|-g\right)}^{2}<\gamma$$

where *g* is the magnitude of the gravity acceleration (1 g or 9.8 m/s^2^) and $\sigma_{a}^{2}$ is the variance of the acceleration signal noise that is used as a scaling factor to make the threshold less sensitive to noise.

#### 2.1.3. Angular Rated Energy Detector (ARED)

On the other hand, the ARED, uses only the angular rate signals as the input. The squared magnitude of the angular rate vector at each instant is compared with a predefined threshold. This can be expressed in the following way

(3) $$V(\text{n})=\frac{1}{\sigma_{\omega }^{2}N}\sum_{k=1}^{N}{\left\|\omega_{k}\right\|}^{2}<\gamma$$

where *ω_k_* is the angular rate vector at instant *k* and $\sigma_{\omega }^{2}$ is the variance of the angular rate noise signal, which is also used as a scaling factor.

#### 2.1.4. Stance Hypothesis Optimal Detector (SHOD)

The SHOD uses both acceleration and angular rate signals. Its goal is to increase the precision of the previous detectors by taking into consideration that there might be instants where human body movement presents angular rate but no acceleration and *vice versa*. The figure of merit used as the input of the classifier is

(4) $$V(\text{n})=\frac{1}{N}\sum_{k=1}^{N}\left(\frac{1}{\sigma_{\text{a}}^{2}}{\left\|{\text{a}}_{k}-g\frac{{\bar{\text{a}}}_{n}}{\left\|{\bar{a}}_{n}\right\|}\right\|}^{2}+\frac{1}{\sigma_{\omega }^{2}}{\left\|\omega_{k}\right\|}^{2}\right)<\gamma$$

#### 2.1.5. Filtered Rectifier Detector (FRD)

The FRD was developed by Veltink [17] as a preprocessing step for a simple classifier of Activities of Daily Life (ADL). The operating principle of the detector is very simple. The frame of the input signal is first high-pass filtered, then rectified and finally low-pass filtered. Therefore, its figure of merit can be expressed as

(5) V(n)=LPE{RECT[HPF(n)]}<γ

In their work the tangential acceleration is used as the input of the detector but, as seen later, the performance of the detector can be improved by using other inputs, such as the magnitude of the acceleration, the magnitude of the angular rate, or a linear combination of both.

### 2.2. Spectrum-Based Methods

Instead of using the time domain to detect possible transitions from inactivity to activity and *vice versa*, now the detectors operate in the frequency domain of the input signals.

#### 2.2.1. Long Term Spectral Detector (LTSD)

The LTSD computes the Long Term Spectral Envelope (LTSE) of the signal. Let *x*(*k*) be the sensor signal which is segmented into frames with a certain degree of overlapping and *X*(*l, n*) its amplitude spectrum for the *l* band at frame n. The N-order long-term spectral envelope can be computed as

(6) $$\text{LTS}E_{N}(l,n)=\text{max}{\left\{X(l,n+j)\right\}}_{j=-N}^{j=+N}$$

The figure of merit used as the input of the classification process for each frame can be obtained by applying

(7) $$V(\text{n})=10\log_{10}\left(\frac{1}{N_{\text{FFT}}}\sum_{l=0}^{N_{\text{FFT}}-1}\frac{\text{LTS}E^{2}(l,n)}{N^{2}(l)}\right)<\gamma$$

where *N_FFT_* = 512 in our case is the resolution of the Fast Fourier Transform and *N*(*l*) is the average noise spectrum magnitude for the band *l*, (*l* = 0, 1,…, *N_FFT_* − 1). For further information about the definition of the LTSD see [21].

#### 2.2.2. Framed Spectrum Detector (FSD)

The FSD is similar to the LTSD, but instead of computing the Long Term Spectral Envelope, it uses the spectrum of each frame in which the input signal is divided. Its expression is as follows

(8) $$V(\text{n})=10\log_{10}\left(\frac{1}{N_{\text{FFT}}}\sum_{l=0}^{N_{\text{FFT}}-1}\frac{X^{2}(l,n)}{N^{2}(l)}\right)<\gamma$$

where again, *N_FFT_* is the resolution of the Fast Fourier Transform, *N*(*l*) is the average noise spectrum magnitude for the band *l* and *X*(*l,n*) is the spectrum of the input signal for the band *l* at frame *n*.

### 2.3. Memory-Based Methods

#### 2.3.1. Memory-Based Theoretic Graph Detector (MBGTD)

The MBGT algorithm is based on computing the distance between two distributions, which are indirectly specified by means of two sample sets. Consider that we have a buffer which is filled with the last *N* sensor readings. Instead of splitting the sample buffer into two equal parts, and testing for difference between them, the MBGT algorithm considers all possible pairs of indices (*i, j*), such that 1≤*i*<*j*≤*N*, which split the sample frame into two adjacent windows *α_ij_*_−1_ and *α_j,N_*, where *α_a,b_* = {*x_a_,x_b_*} is a window that contains all samples from index *a* to index *b, i.e.*, the starting points of the first and second window respectively. Once we have divided the frame into two sub-windows, we can compute the average Euclidean distance between two points included in the pair (*i,j*) applying the following expression

(9) $$C_{i,j}=\frac{\sum_{k=i}^{j-1}\sum_{l=j}^{N}d_{k,l}}{\left(j-i)(N-j+1\right)}$$

where *d_k,l_* is the Euclidean distance between points *k* and *l*.

The overall figure of merit of the detection algorithm is the maximum *C_i,j_* computed over all the possible frame splits, which is

(10) $$V_{\text{MBGTD}}=\underset{1\leq i<j\leq N}{\max}C_{i,j}<\gamma$$

Further information about the algorithm and about how to implement it so its complexity is suitable for practical applications can be found in [25].

#### 2.3.2. Memory-Based CUSUM Detector (MBCD)

The Memory-Based Cumulative Sum Detector is a variation of the well-known Cumulative Sum (CUSUM) algorithm first proposed in [26] and explained in depth in [27]. The CUSUM algorithm accumulates the log-likelihood of the current reading with respect to the distributions, before (*p_θ_*_0_(*x_i_*)) and after (*p_θ_*_1_(*x_i_*)), which is the hypothesized change point. The procedure is as follows

(11) $$\begin{array}{cc} g_{k}=S_{k}-m_{k}, & S_{k}=\sum_{i=1}^{k}s_{i}\\ s_{i}=\ln\frac{p_{\theta_{1}}(x_{i})}{p_{\theta_{0}}(x_{i})}, & m_{k}=\underset{1\leq j\leq k}{\min}S_{j} \end{array}$$

The change point will be detected when *g_k_* ≤ *h* where *h* is an empirically set threshold.

However, the CUSUM algorithm can only be applied when both the distributions (*p_θ_*_0_(*x_i_*)) and (*p_θ_*_1_(*x_i_*)) are known. The MBCD solves this drawback by estimating both pre-change and post-change distributions via Parzen kernel density estimates [28] by using the following expression (included here forclarity),

(12) $${\overset{⌢}{p}}_{\theta }(x_{k})=\frac{1}{N{\left(2\lambda^{2}\pi \right)}^{\frac{1}{2}}}\sum_{i=1}^{N}e^{\frac{1}{2}{\left(\left\|x_{1}-x_{k}\right\|/\lambda \right)}^{2}}$$

where *λ* stands for the standard deviation of the distribution and *N* is the total number of samples included in the frame.

Once we know how to estimate the distributions of each one of the sub-windows, we can proceed to compute the log-likelihood ratio as follows

(13) $$S_{i.j}=\overset{N}{\underset{l=j}{\text{∑}}}\log\frac{\frac{1}{N-j+1}\sum_{k=j}^{N}w_{l,k}}{\frac{1}{j-1}\sum_{k=i}^{j-1}w_{l,k}}$$

where *w_l,k_* is a kernel weight for the pair of samples (*x_l_, x_k_*) computed using Parzen's approximation.

The general figure of merit of the algorithm is

(14) $$V_{\text{MBCD}}=\underset{1\leq i<j\leq N}{\max}S_{i,j}<\gamma$$

Further information about the algorithm and how to reduce its computational complexity can also be found in [25].

#### 2.3.3. Workflow of the Algorithms

All the presented algorithms have been implemented following the structure which is explained by the steps mentioned below.

- Set input parameters of the algorithm.
- Algorithm starts a swipe, using a sliding window, through the input signal.
- For every signal frame, compute the resultant value of the figure of merit by applying Equations (1–5), (7), (8), (10), (14) accordingly. Save computed value in a vector that grows in length with the iterations.
- Compare figures of merit obtained for each one of the aforementioned methods with the predefined threshold. For every instant *k*, if the value of the figure of merit is lower than the threshold, we will mark the instant as “static” and a 0 is added to a marker vector. On the other hand, if the value of the vector is equal or higher than the threshold, the instant will be marked as “active” and a value of 1 is added to the marker vector. At the end of the application of every algorithm we will have a binary marker vector what will be used for performance evaluation.

Finally, the following list clarifies the corresponding inputs and outputs of every presented algorithm:

- Input:
  – Signal to be analyzed:
    * Acceleration (X, Y and Z axes): AMVD and AMD.
    * Angular Rate (X, Y and Z axes): ARED.
    * Acceleration and Angular rate (X, Y and Z axes): SHOD.
    * Flexible input: FRD, LTSD, FSD, MBGTD and MBCD.
  – Window length (size of sliding window): AMVD, AMD, ARED, SHOD, LTSD, FSD, MBGTD and MBCD
  – Threshold (empirically predefined): AMVD, AMD, ARED, SHOD, FRD, LTSD, FSD, MBGTD and MBCD.
  – Shift (sliding window overlapping): LTSD and FSD.
- Output:
  – Figure of merit.
  – Binary activity marker (computed by comparing the figure of merit with the predefined threshold).

## 3. Experiments

Once the detectors are implemented we need to design a comparative study that computes different statistic parameters to determine the performance of each algorithm. Such a comparative study is divided in two parts. The first part includes simulations derived from the application of the detectors on a large set of synthesized signals, and the second part aims to complete the study by applying the algorithms on real datasets gathered from inertial sensors.

### 3.1. Simulations

The main goal of the theoretical simulations is to apply the algorithm over a very large set of signals, since this will alow the computed performance parameters to have statistic significance. Specifically, we will be calculating the Accuracy and Correlation coefficient of the resultant activity marker with respect to the actual activity marker. The actual markers are obtained by visually inspecting each one of the gathered acceleration and angular rate signals and hand labeling the starting and ending points of each activity period. This is done by averaging the observed starting and ending points. Due to the cumbersomeness and almost impracticality of carrying out such a procedure over a large set of signals, we decided to design a synthesizer that is able to mimic signals coming out of an accelerometer and a gyroscopic sensor. The synthesizer is designed not only to avoid the hand-labeling procedure but to be able to generate large data sets as gathering many real signals is very time consuming. Therefore, the synthesizer will also generate the marker with the actual starting and ending points of each activity period so we do not have to label them manually.

#### 3.1.1. Set-Up

At the start of the simulations we need to generate the synthetic signals and to that purpose we use the signal synthesizer. The signal synthesizer has been built to generate acceleration-like and angular rate-like signals coming from five different basic activities: walking, sitting on a chair and standing up, laying on a bed and standing up, running and jumping. Two more general activities have been implemented. The first one includes no acceleration and shows a constant angular rate and the second one includes no angular rate and shows a constant acceleration period. Although this may look like an unrealistic activity, there exist instants of time where this may happen. Thus, we have included them to ensure that the detector is as much robust as possible. The intensity of each activity, *i.e.*, frequency, amplitude, and also the length of each activity period are set randomly every time the synthesizer is called. The sensing axis that we want to be parallel to the gravity vector can also be set. In addition, random noise is added to the signals in order to get a better approximation of real sensor signals. The magnitude of the acceleration is set to be 1*g* at every static instant. The acceleration and angular rate ranges can also be set according to the level of expected acceleration that will be present in the exercise that we are simulating, and also to simulate similar ranges to those of commercial MEMS inertial sensors.

**Figure 2.**
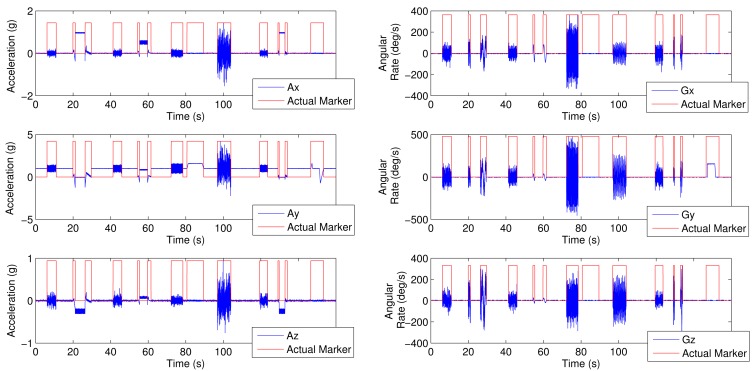
Acceleration, angular rate synthesized signals and activity marker. Activity sequence: walking, laying-standing up, walking, sitting-standing up, running, no angular rate, jumping, walking, laying-standing up, no acceleration.

Figure 2 shows the synthesized signals for the following activity sequence: walking, laying down-standing up, walking, sitting down-standing up, running, no angular rate, jumping, walking, laying down-standing up, no acceleration. The depicted binary activity marker is normalized to the signal magnitude to allow visibility.

#### 3.1.2. Monte Carlo Simulation

**Figure 3.**
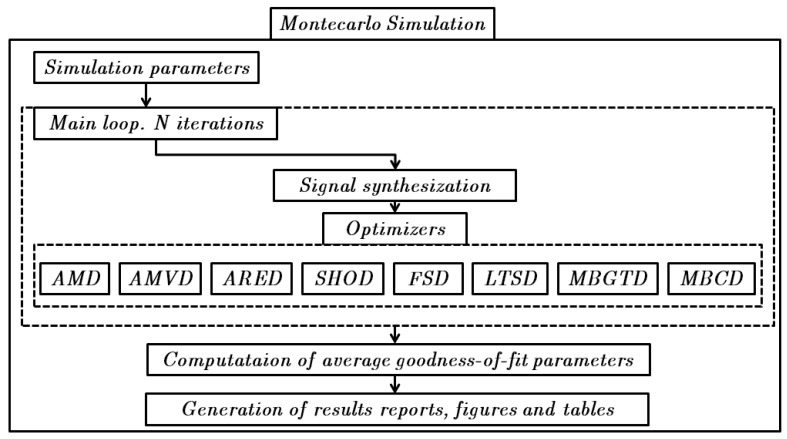
Theoretical simulation diagram. A Monte Carlo simulation is performed to ensure statistical robustness.

Once the signal synthesizer is set, a Monte Carlo simulation of *N* repetitions can be performed. At every repetition a new set of signals is synthesized. Then, an optimization routine based on a grid search procedure is called for every algorithm. We opted for a grid search procedure since we observed that convergence of the objective functions depended highly on the initial values of the parameters to be optimized and this was causing the optimizers to stop in local minima that were far from the optimal values. The optimizer performs a sweep through the different parameters of each method, for example, window length and threshold for magnitude-based and memory-based methods and window length, frame shift and threshold for the spectrum-based methods. For every parameter configuration, the accuracy and correlation coefficient are computed. After the sweep, we extract the maximum values of the statistics and also store the value of the parameters for which they maximize. At the end of the Monte Carlo simulation, we obtain the average value of every statistic and the average value of the optimal configuration parameters for each one of the eight methods. Figure 3 depicts a diagram showing the steps to be followed during the theoretical simulation.

Spectrum-based and memory-based methods can be computed using different combinations of sensor inputs. We have used four different combinations: the magnitude of the acceleration; the magnitude of the angular rate; and the sum and product of both acceleration and angular rate magnitudes. Proceeding this way, we will be able to determine which of the sensor combinations offers the best performance.

**Figure 4.**
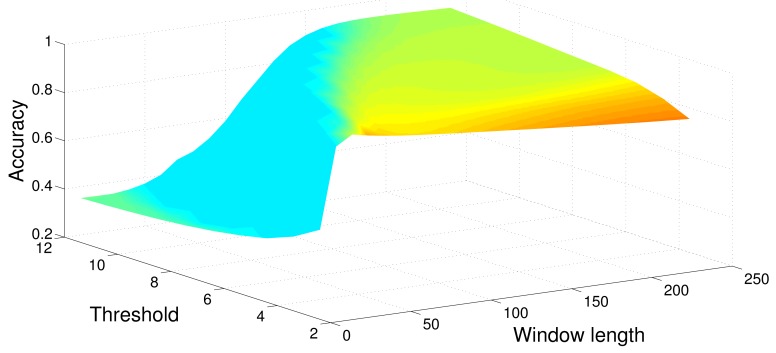
Parameter optimization. Sweep of window length and threshold values to find maximum accuracy (MBGTD).

Tables 1–6 show the average Accuracy and Correlation coefficient values, as well as the associated parameter values, for each one of the detection methods put into a Monte Carlo simulation of *N* = 500 runs. Therefore, all the eight methods have been tested using a set composed of 500 synthetic signals. Figure 4 shows the average Accuracy values obtained from the optimization procedure, when sweeping values of window size and threshold, searching for maximum accuracy.

### 3.2. Real Datasets

In order to check the theoretical results obtained in the simulations we have gathered a set of signals using two Wagyromag Inertial Measurement Units (IMUs), that we previously designed [29]. Wagyromag includes the following sensors;

An Analog Devices MEMS ADXL335 triaxial accelerometer [30]. It has a frequency response ranging from 0.5 Hz to 1, 600 Hz for X and Y axes and from 0.5 Hz to 550 Hz for Z axis. It measures the acceleration in a ±3 g dynamic range. It has a sensibility of 300 mV/g and the offset variations are lower than ±1 mg/°C.

Two ST Microelectronics MEMS Coriolis vibratory gyroscopes are employed to sense angular velocity: LPR550AL [31] (axes X and Y) and LY550ALH [32] (axis Z). It is one of the low cost MEMS gyros offering the lowest temperature drift coefficient (the typical variation of the offset is 0.08°/s/°C and the typical sensibility variation is 0.03%/°C). Both sensors have a bandwidth of 140 Hz and measure the angular rate in a ±500°/s range.

A Honeywell HMC5843 triaxial magneto-resistive sensor [33]. It offers a selectable dynamic range between ±0.7 and ±6.5 *Gauss*. This device is suited for measuring position with respect to the magnetic north with a precision of ±0.5°. It measures the magnetic field from tens of micro-gauss to 6 gauss.

A Microchip MCP9700A analog temperature sensor [34] with a temperature range from *−*40 °C to +125 °C. The accuracy is stated with a maximum of ±2 °C (0 °C to +70 °C). It is included to add temperature compensation to the calibration procedure applied to the sensors.

**Figure 5.**
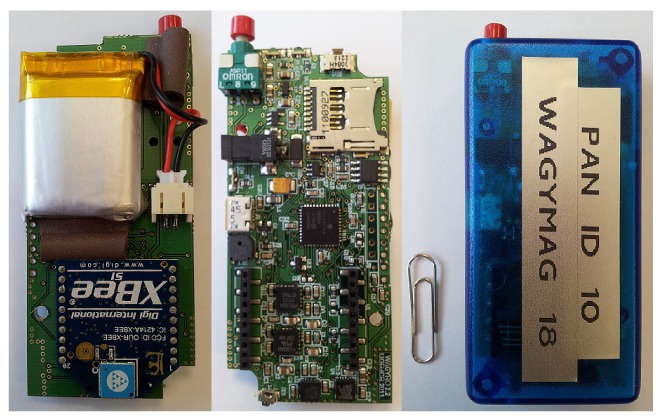
Internal (left and center) and external (right) appearance of Wagyromag, the employed IMU to gather inertial data.

Figure 5 shows the internal and external appearance of Wagyromag, our IMU prototype.

#### 3.2.1. Set-Up

Three male healthy subjects (179.33±4.04 cm, 72.33±7.09 kg, 25±1 years) wearing two Wagyromag units placed at the hip and the ankle respectively performed twice a circuit composed of the following activities: walk 20 m, stop, sit down-stand up, stop, run 20 m, stop, jump 5 times, stop, and lay downstand up. A total of 96 signals were gathered (3 acceleration axes + 3 angular rate axes)×2 IMUs×4 subjects× 2 runs) and used as the input for all detection algorithms.

The (in)activity markers were set manually by visually inspecting the gathered signals.

#### 3.2.2. Optimization of Parameters

Like in the theoretical simulations, an analogous optimization procedure was carried out using the real dataset in order to obtain the average maximum Accuracy and Correlation coefficient values and their associated algorithm configuration parameters. By doing this we aimed to verify those results previously obtained from the theoretical simulation and check for possible differences. Tables 7–12 show the average values obtained when the detectors are applied to the 96 signals.

**Figure 6.**
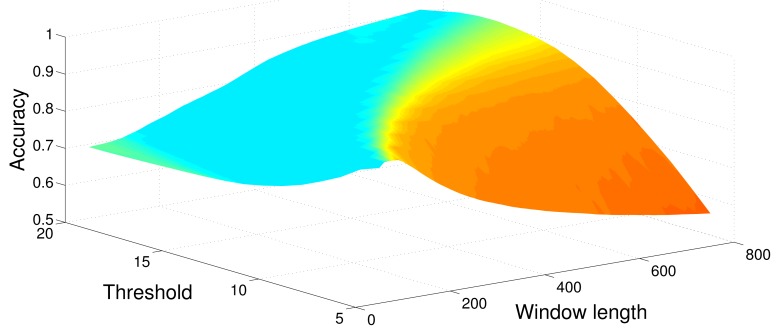
Parameter optimization. Sweep of window length and threshold values to find maximum accuracy (MBGTD). Real signals.

**Figure 7.**
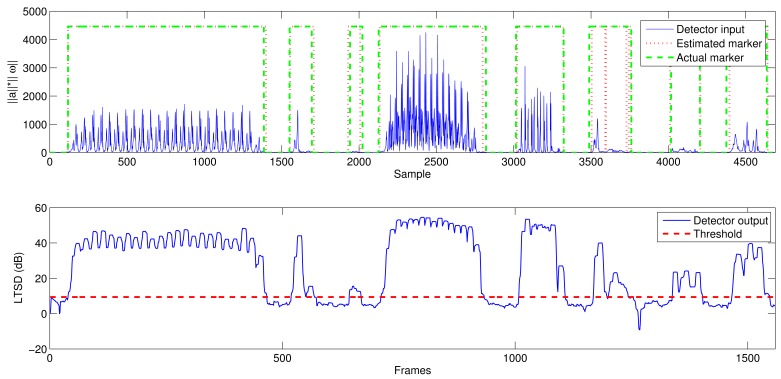
Input (product of acceleration and angular rate magnitude) and output (vector of characteristics and marker) of the LTSD. Real signals.

**Figure 8.**
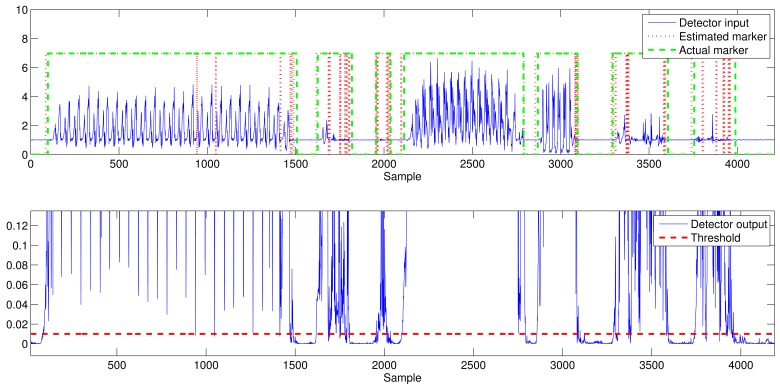
Input and output (vector of characteristics and marker) of the AMVD. Real signals.

Figure 6 shows the average output of the optimization process when maximizing the accuracy and using the MBGTD. Figures 7 and 8 show the input (product of acceleration and angular rate magnitudes) and output (figure of merit) of the AMVD and the LTSD for a set of gathered signals when subject number 1 is following the activity circuit wearing the IMU at the ankle. Binary activity markers have been normalized to the input amplitude in order to allow visibility in the same plot.

## 4. Results Discussion

We now proceed to discuss the results obtained in the experiments we carried out. In the first part of the section we will analyze and compare all the tested algorithms between them. Additionally, in the second part, we compare our results to those obtained in other works present in the literature.

### 4.1. Results of Our Experiments

When analyzing the results thrown by the theoretical simulations using magnitude-based methods (Table 1) we observe that SHOD has the highest Accuracy and Correlation coefficient and also the highest AUC. Since SHOD uses both acceleration and angular rate signals its detection rate is less affected by non-accelerated or non-spinning movements. AMD classifies second in the performance evaluation even when it is not able to detect the non-accelerated movements as it solely relies on the acceleration signals to carry out the detection. AMVD shows the poorest performance of magnitude-based methods. This is due to the fact that when there is an abrupt change in the signal, the variance value will be high, which causes the detector to prematurely detect the transition from a static state to an active state. Analogously, it also prematurely detects the transition from an active state to a static state. These shifts in the estimated marker are the main cause of its poor performance. FRD has a very poor performance when using just the acceleration magnitude. This happens because abrupt changes are smoothed by the filtering process and, therefore, large shifts are introduced at the starting and ending points of each activity period.

On the other hand, the performance of the spectrum methods is somewhere between the performance of the AMVD and the ARED. Amongst them, FSD using the product of the acceleration and the angular rate magnitudes as the input does the best in terms of accuracy. This is due to the fact that the product of the magnitudes will increase the resultant amplitude of activity periods leading to values much higher than the threshold, *i.e.*, detectable values. The LTSD method has a worse decision rate as it is designed to work under conditions where the SNR is low, *i.e.*, the sensor signals present large noise, which is not the case for our synthesized signals.

Memory-based methods are thought for detecting any abrupt change in signals. This means they also detect changes during active periods. For example, if the subject starts to run faster, the resultant inertial signals will have a larger amplitude and frequency and the figure of merit of the detector will have a higher output. This can be a drawback because if the intensity change during an activity period is very radical, which is similar to a change from inactivity to activity, the detector may wrongly detect the change as a transition from activity to inactivity.

**Figure 9.**
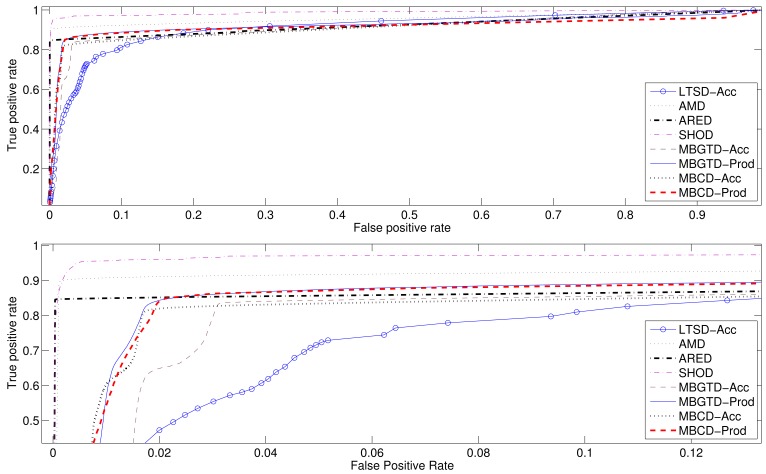
ROC curves computed for the eight best methods. Synthesized signals. Complete curves (up), zoomed curves (down).

**Figure 10.**
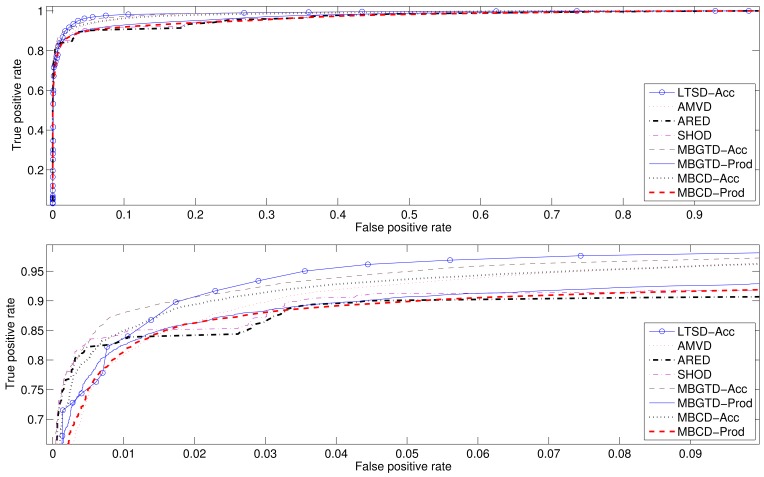
ROC curves computed for the eight best methods. Real signals. Complete curves (up), zoomed curves (down).

In addition to the Accuracy and the Correlation coefficient, we have also computed the ROC curves and Area Under Curve (AUC) values to follow the standards used to compare detectors and to ease the performance classification of all tested methods. Figures 9 and 10 show the average ROC curves for the best eight methods when applied to synthesized signals and gathered real signals, respectively. Tables 13 and 14 show the computed AUC values for each one of the algorithms when they are applied to, synthesized and real signals, respectively. As we can see, those methods having high Accuracy rates also present high AUC values. A high AUC value means that the detection algorithm has low False Positive rates when the True Positive rate is high, which is the desired behavior of a classifier.

In terms of parameter configuration, we would prefer a shorter window length if we are monitoring movement in real time. Most methods have an optimal window size of around 10 samples which is an adequate latency for real time applications. Only AMD has a latency of 80 samples until it is able to start the detection procedure. This translates to a continuous delay of almost two seconds during the whole monitoring session when we use an IMU having a sampling frequency of 50 Hz like the one we used in the present work.

Now, if we look at the results when real signals are used, we can see that the effectiveness of the spectrum and memory-based methods has improved. LTSD using just the acceleration magnitude as input has the best accuracy of all tested methods (0.9711 ± 0.0072). Acceleration signals gathered using the IMU showed a slightly larger noise than the synthesized signals. This may have caused the performance increase of spectrum methods. Both MBGTD and MBCD present a raise of 3% in the accuracy rate, as the subjects did not perform abrupt changes of intensity while running or walking, which decreased the rate of false changes from activity to inactivity. Alternatively, the raise in the general performance of spectrum and memory-based methods could also be a result of the lower number of real signals that were used to run the tests compared to the number of synthesized signals.

AMD presents a lower performance when monitoring real signals as the zero-crossing-rate was higher than in the theoretical case. Its poorer performance is caused by the high amount of instants where the acceleration crosses the zero level. After computing the magnitude of the acceleration, the values corresponding to zero-crossing instants will still be zero or close to zero; they will be below the threshold and the instant will be erroneously classified as “static”. AMVD does better as the transitions from states in real signals are smoother than in synthesized signals.

Computation times of both memory-based and spectrum-based methods are larger than magnitude based methods when executed in a regular computer. Difference in computation time can be much higher if the algorithms are implemented in processors embedded in mobile devices or IMUs. This may lead to unacceptable delays in real time monitoring applications. However, this is not a problem if signals are being processed both online or offline in a regular computer. Implementation of magnitude-based methods such as SHOD should be considered when using devices that have low computation power.

### 4.2. Comparison with Results in Literature

Our main contribution in this work is the proposal of new algorithms to detection of human body (in)activity periods using inertial sensors, as well as other existing detection algorithms that had not been applied to this field yet. We have also extended the work in [20] by using a larger amount of algorithms and signals to increase the statistical significance of the results.

We have obtained similar results for the methods tested in [20] since SHOD has revealed to be superior to the rest of magnitude-based methods. To our knowledge, one of the first methods developed to detect (in)activity using inertial sensors was presented in [17]. We have shown that, while their method has acceptable rates of accuracy (∼85%), the subsequently developed magnitude-based algorithms, as well as our proposals, outperform it.

Not all works presenting detection methods contain an explicit performance study, as in most cases the algorithms were developed as a part of a more complex system with different goals (activity classification, human body positioning algorithms, inertial navigation, *etc.*). Therefore, it is not easy to compare our results with those obtained by them.

In summary, average maximum accuracy rates and correlation coefficients between the actual activity markers and the markers computed by the algorithms have been presented, together with the optimal configuration parameters, in Tables 1–6 and 7–12, for synthesized and real signals respectively. ROC curves of the best methods, as well as their associated AUC values, have been revealed in Figures 9 and 10 and Tables 13 and 14.

## 5. Conclusions and Future Work

The main motivation of the presented work was to help readers wishing to implement an (in)activity detector for human body movement monitoring (and also other applications such as inertial navigation) to choose and appropriate algorithm. To do so, we have carried out a rigorous and complete comparative study between different algorithms that have been applied in recent literature to detect (in)activity periods in human body motion by means of inertial sensors. To extend the study, we have proposed and tested other methods that are being applied to detect abrupt changes in signals in different applications (industrial processes, voice detection, *etc.*) that had never been applied to the motion detection field.

Discrimination of (in)activity periods is of critical importance in inertial navigation algorithms so the Zero Velocity Updates (ZUPT) can be computed. It is also a very important preprocessing step for inertial-based human activity classifiers since it helps to divide the signals into periods that are later analyzed.

Along the paper, we have presented a comparative study among different magnitude-based algorithms provided in literature, such as the Acceleration Moving Variance Detector (AMVD), the Acceleration Magnitude Detector (AMD), the Angular Rate Energy Detector (ARED), the Stance Hypothesis Optimal Detector (SHOD), and the Filtered Rectifier Detector (FRD). The study presented in [20] has been completed by using a larger data set of theoretical signals. Moreover, a new approach has been tested. It includes spectrum-based algorithms such as the Framed Spectrum Detector (FSD) and the Long Term Spectral Detector (LTSD) and memory-based algorithms such as the Memory-Based Graph Theoretical Detector and The Memory-Based Cumulative Sum Detector (MBCD). The objective was to carry out a statistically robust comparison. To do so, we developed an acceleration and angular rate signal synthesizer that mimics the output of a triaxial accelerometer and a triaxial gyroscope when a subject is performing basic activities such as walking, running, laying, sitting, standing up and jumping. The theoretical tests show that SHOD is the method with the highest accuracy rate achieving ROC values higher than 0.96. In contrast, tests applied using real signals place LTSD, which uses the magnitude of the acceleration as input, as the best detector with an accuracy rate of 0.9711 ± 0.0072. This method is closely followed by FSD-Acc. achieving a correlation coefficient of 0.9302 ± 0.0155 and an accuracy rate of 0.9702 ± 0.0064.

The use of SHOD is strongly recommended when the system has a reduced computation power and/or when lower delay is preferred over higher precision. Alternatively, LTSD is the best option if movement is being analyzed using a powerful computer and/or in an offline way.

Future work will focus on improving the quality of the signal synthesizer by increasing the resemblance between the synthesized signals and the real ones as well as including other activities of daily life in its repertoire. Other existent abrupt change detection algorithms will also be tested over a larger set of real signals to increase the statistical significance of the obtained results.

## Figures and Tables

**Table 1.** Results of the Monte Carlo simulation (*N* = 500). Synthesized signals. Average Accuracy, Correlation coefficient and associated parameters (Magnitude methods without flexible input).

**Optimal value**	**AMVD**	**AMD**	**ARED**	**SHOD**
Accuracy	0.8741 ± 0.0181	0.9641 ± 0.0087	0.9431 ± 0.0136	**0.9817 ± 0.0124**
Correlation coeff.	0.7137 ± 0.0360	0.9205 ± 0.0175	0.8752 ± 0.0270	**0.9592 ± 0.0269**
Window length	26.065 ± 1.1011	96.9560 ± 9.8260	21.9300 ± 1.5849	10.7556 ± 1.3007
Threshold	0.0188 ± 0.0049	0.0008 ± 0.0002	2.3712 ± 2.1621	1.3426 ± 0.2898

**Table 2.** Results of the Monte Carlo simulation (*N* = 500). Synthesized signals. Average Accuracy, Correlation coefficient and associated parameters (Framed Spectrum Detector).

**Optimal value**	**FSD-Acc.**	**FSD-Ang.**	**FSD-SUM.**	**FSD-PROD.**
Accuracy	0.9395 ± 0.0155	0.9344 ± 0.0153	0.9330 ± 0.0351	**0.9470 ± 0.0441**
Correlation coeff.	0.8639 ± 0.0331	0.8534 ± 0.0311	0.8520 ± 0.058906	**0.8835 ± 0.0788**
Window length	18.0836 ± 1.8278	17.3496 ± 1.8112	13.0636 ± 0.9983	9.5292 ± 1.7607
Threshold	2.6441 ± 0.6272	13.8640 ± 3.0741	10.4764 ± 2.6726	8.3344 ± 2.8890
Shift	15.1304 ± 2.2318	16.9824 ± 0.9055	13.1048 ± 0.4916	7.2364 ± 2.9423

**Table 3.** Results of the Monte Carlo simulation (*N* = 500). Synthesized signals. Average Accuracy, Correlation coefficient and associated parameters (Long Term Spectral Detector).

**Optimal value**	**LTSD-Acc.**	**LTSD-Ang.**	**LTSD-SUM**	**LTSD-PROD.**
Accuracy	0.9252 ± 0.0179	0.9150 ± 0.0926	**0.9355 ± 0.0138**	0.9318 ± 0.0884
Correlation coeff.	0.8328 ± 0.0388	0.8209 ± 0.15256	**0.8556 ± 0.0282**	0.8585 ± 0.1266
Window length	14.6012 ± 0.7580	5.2376 ± 0.8156	3.3848 ± 0.2816	11.3200 ± 0.3639
Threshold	4.9140 ± 1.7976	17.3252 ± 3.0170	16.1172 ± 3.6251	8.7112 ± 1.7633
Shift	1.6348 ± 0.7648	1.3936 ± 0.5699	1.2744 ± 0.5312	1.7776 ± 0.7616

**Table 4.** Results of the Monte Carlo simulation (*N* = 500). Synthesized signals. Average Accuracy, Correlation coefficient and associated parameters (Memory Based Graph Theoretic Detector).

**Optimal value**	**MBGTD-Acc.**	**MBGTD-Ang.**	**MBGTD-SUM.**	**MBGTD-PROD.**
Accuracy	0.9243 ± 0.0139	0.9114 ± 0.0179	0.9115 ± 0.0179	**0.9349 ± 0.0159**
Correlation coeff.	0.8295 ± 0.02813	0.8040 ± 0.0345	0.8041 ± 0.0345	**0.85312 ± 0.03532**
Window length	12.8088 ± 3.4643	5.9932 ± 3.4687	5.9900 ± 3.4701	9.7260 ± 3.7743
Threshold	1.1286 ± 0.7215	84.9760 ± 79.7386	84.8680 ± 79.275	151.8720 ± 81.4212

**Table 5.** Results of the Monte Carlo simulation (*N* = 500). Synthesized signals. Average Accuracy, Correlation coefficient and associated parameters (Memory Based CUSUM Detector).

**Optimal value**	**MBCD-Acc.**	**MBCD-Ang.**	**MBCD-SUM.**	**MBCD-PROD.**
Accuracy	0.9257 ± 0.0145	0.9098 ± 0.0180	0.9100 ± 0.0180	**0.9373 ± 0.0168**
Correlation coeff.	0.8339 ± 0.0289	0.80002 ± 0.034774	0.80066 ± 0.034845	**0.85877 ± 0.037214**
Window length	8.4584 ± 3.1602	7.6108 ± 2.4729	6.0644 ± 2.1265	11.1192 ± 3.2323
Threshold	1.749e−6 ± 5.389e−7	0.1117 ± 0.0179	0.0925 ± 0.0182	0.1068 ± 0.0267

**Table 6.** Results of the Monte Carlo simulation (*N* = 500). Synthesized signals. Average Accuracy, Correlation coefficient and associated parameters (Filtered Rectifier Detector).

**Optimal value**	**FRD-Acc.**	**FRD-Ang.**	**FRD-SUM.**	**FRD-PROD.**
Accuracy	0.7921 ± 0.0178	0.7608 ± 0.0207	0.7610 ± 0.0207	**0.7944 ± 0.0223**
Correlation coeff.	0.5228 ± 0.0389	0.4823 ± 0.0464	0.4825 ± 0.0463	**0.5362 ± 0.0509**
Threshold	0.0100 ± 0.0070	0.1720 ± 0.2862	0.1780 ± 0.2990	0.1680 ± 0.2777

**Table 7.** Algorithms applied to real signals. Average Accuracy, Correlation coefficient and associated parameters (Magnitude methods without flexible input).

**Optimal value**	**AMVD**	**AMD**	**ARED**	**SHOD**
Accuracy	**0.9529 ± 0.0113**	0.8875 ± 0.0196	0.9418 ± 0.0185	0.9447 ± 0.0236
Correlation coeff.	**0.8899 ± 0.02536**	0.7610 ± 0.0411	0.8678 ± 0.0381	0.8730 ± 0.0473
Window length	16.7333 ± 2.3851	86.2000 ± 36.2165	8.5167 ± 6.5721	19.6167 ± 9.2617
Threshold	0.0173 ± 0.0106	0.0011 ± 0.0006	38.3250 ± 26.9008	2.3995 ± 1.1856

**Table 8.** Algorithms applied to real signals. Average Accuracy, Correlation coefficient and associated parameters (Framed Spectrum Detector).

**Optimal value**	**FSD-Acc.**	**FSD-Ang.**	**FSD-SUM.**	**FSD-PROD.**
Accuracy	**0.9702 ± 0.0064**	0.9533 ± 0.0194	0.9479 ± 0.0151	0.9515 ± 0.0162
Correlation coeff.	**0.9302 ± 0.0155**	0.8918 ± 0.0420	0.8804 ± 0.0359	0.8886 ± 0.0385
Window length	20.2000 ± 9.2214	16.4500 ± 4.8910	13.5167 ± 2.5943	14.3000 ± 6.3390
Threshold	3.2433 ± 1.3441	5.0667 ± 2.3935	5.0583 ± 2.72227	5.2917 ± 2.6446
Shift	18.6667 ± 7.0711	15.9667 ± 2.6592	9.4667 ± 1.4477	9.5667 ± 2.5293

**Table 9.** Algorithms applied to real signals. Average Accuracy, Correlation coefficient and associated parameters (Long Term Spectral Detector).

**Optimal value**	**LTSD-Acc.**	**LTSD-Ang.**	**LTSD-SUM.**	**LTSD-PROD.**
Accuracy	**0.9711 ± 0.0072**	0.9682 ± 0.0096	0.9523 ± 0.0591	0.9670 ± 0.0122
Correlation coeff.	**0.9318 ± 0.0186**	0.9261 ± 0.0228	0.9165 ± 0.0428	0.9264 ± 0.0225
Window length	13.8500 ± 6.4327	5.1167 ± 2.4056	4.6833 ± 0.8023	10.7500 ± 2.5498
Threshold	5.4167 ± 1.9185	8.9167 ± 3.0781	8.7083 ± 2.3045	9.0500 ± 2.9711
Shift	2.4500 ± 0.6390	2.2333 ± 0.8628	1.9167 ± 0.6640	2.5833 ± 1.1134

**Table 10.** Algorithms applied to real signals. Average Accuracy, Correlation coefficient and associated parameters (Memory Based Graph Theoretic Detector).

**Optimal value**	**MBGTD-Acc.**	**MBGTD-Ang.**	**MBGTD-SUM.**	**MBGTD-PROD.**
Accuracy	**0.9626 ± 0.0071**	0.9452 ± 0.0120	0.9452 ± 0.0121	0.9468 ± 0.0109
Correlation coeff.	**0.9125 ± 0.0186**	0.8632 ± 0.0383	0.8634 ± 0.0384	0.8670 ± 0.0359
Window length	13.1833 ± 4.5759	13.6833 ± 5.0705	13.6167 ± 5.0182	13.6500 ± 5.0040
Threshold	1.5467 ± 0.7218	454.0000 ± 285.2036	447.333 ± 279.4448	453.5000 ± 280.7149

**Table 11.** Algorithms applied to real signals. Average Accuracy, Correlation coefficient and associated parameters (Memory Based CUSUM Detector).

**Optimal value**	**MBCD-Acc.**	**MBCD-Ang.**	**MBCD-SUM.**	**MBCD-PROD.**
Accuracy	**0.9576 ± 0.0080**	0.9414 ± 0.0164	0.9414 ± 0.0165	0.9434 ± 0.0154
Correlation coeff.	**0.9010 ± 0.0153**	0.8588 ± 0.0465	0.8583 ± 0.0469	0.8635 ± 0.0429
Window length	12.6167 ± 4.1869	15.2500 ± 6.4345	15.0500 ± 6.8720	14.5167 ± 6.6832
Threshold	3.468e-6 ± 2.049e-6	0.3588 ± 0.1731	0.3551 ± 0.1746	0.4346 ± 0.1690

**Table 12.** Algorithms applied to real signals. Average Accuracy, Correlation coefficient and associated parameters (Filtered Rectifier Detector (FRD)).

**Optimal value**	**FRD-Acc.**	**FRD-Ang.**	**FRD-SUM.**	**FRD-PROD.**
Accuracy	0.8136 ± 0.0282	**0.8417 ± 0.0218**	0.8414 ± 0.0218	0.8248 ± 0.0305
Correlation coeff.	0.5754 ± 0.0616	**0.6319 ± 0.0657**	0.6313 ± 0.0653	0.5878 ± 0.0703
Threshold	0.0055 ± 0.0077	0.1508 ± 0.0928	0.1566 ± 0.0899	0.1558 ± 0.0903

**Table 13.** Area Under Curve (AUC) computed out of ROC curves obtained from application of algorithms on synthesized signals. Number in brackets indicates overall position in performance comparison.

AUC	**AMVD** 0.8778 (12)	**AMD** 0.9576 (2)	**ARED** 0.9256 (3)	**SHOD** 0.9880 (1)
AUC	**FSD-Acc.** 0.8897 (11)	**FSD-Ang.** 0.7909 (18)	**FSD-Sum** 0.7722 (21)	**FSD-Prod** 0.7422 (24)
AUC	**LTSD-Acc.** 0.9127 (7)	**LTSD-Ang.** 0.7468 (23)	**LTSD-Sum** 0.8940 (10)	**LTSD-Prod** 0.8959 (9)
AUC	**MBGTD-Acc.** 0.9090 (8)	**MBGTD-Ang.** 0.7478 (22)	**MBGTD-Sum** 0.8648 (15)	**MBGTD-Prod** 0.9250 (4)
AUC	**MBCD-Acc.** 0.9133 (6)	**MBCD-Ang.** 0.8655 (13)	**MBCD-Sum** 0.8650 (14)	**MBCD-Prod** 0.9172 (5)
AUC	**FRD-Acc.** 0.8451 (16)	**FRD-Ang.** 0.7787 (19)	**FRD-Sum** 0.7786 (20)	**FRD-Prod** 0.8177 (17)

**Table 14.** Area Under Curve (AUC) computed out of ROC curves obtained from application of algorithms on real signals. Number in brackets indicates overall position in performance comparison.

AUC	**AMVD** 0.9847 (2)	**AMD** 0.9239 (13)	**ARED** 0.9662 (8)	**SHOD** 0.9695 (6)
AUC	**FSD-Acc.** 0.8850 (14)	**FSD-Ang.** 0.7183 (21)	**FSD-Sum** 0.7083 (22)	**FSD-Prod** 0.6153 (24)
AUC	**LTSD-Acc.** 0.9870 (1)	**LTSD-Ang.** 0.6965 (23)	**LTSD-Sum** 0.8610 (18)	**LTSD-Prod** 0.9284 (12)
AUC	**MBGTD-Acc.** 0.9798 (4)	**MBGTD-Ang.** 0.8211 (20)	**MBGTD-Sum** 0.9536 (10)	**MBGTD-Prod** 0.9726 (5)
AUC	**MBCD-Acc.** 0.9845 (3)	**MBCD-Ang.** 0.9557 (9)	**MBCD-Sum** 0.9431 (11)	**MBCD-Prod** 0.9666 (7)
AUC	**FRD-Acc.** 0.8702 (15)	**FRD-Ang.** 0.8690 (16)	**FRD-Sum** 0.8689 (17)	**FRD-Prod** 0.8461 (19)

## References

[1] Fuentes L., Jiménez D., Pinto M. (2006). Development of ambient intelligence applications using components and aspects. J. Univ. Comput. Sci.

[2] Zhou H., Hu H. (2008). Human motion tracking for rehabilitation—A survey. Biomed. Signal Proces.

[3] Luinge H.J., Veltink P.H. (2004). Inclination measurement of human movement using a 3-D accelerometer with autocalibration. IEEE Trans. Neural Syst. Rehabil.

[4] Favre J., Jolles B.M., Aissaoui R., Aminian K. (2007). Ambulatory measurement of 3D knee joint angle. J. Biomech.

[5] Beiji Z., Shu C., Cao S., Umugwaneza M. (2009). Automatic reconstruction of 3D human motion pose from uncalibrated monocular video sequences based on markerless human motion tracking. Pattern Recogn.

[6] Tao Y., Hu H., Zhou H. (2007). Integration of vision and inertial sensors for 3D arm motion tracking in home-based rehabilitation. Int. J. Robot. Res.

[7] Cai Y. (2010). Mobile intelligence. J. Univ. Comput. Sci.

[8] Tentori M., Favela J., Gonzalez V.M. (2006). Quality of Privacy (QoP) for the design of ubiquitous healthcare applications. J. Univ. Comput. Sci.

[9] Lemkin M., Boser B.E. (1999). A three-axis micromachined accelerometer with a CMOS position-sense interface and digital offset-trim electronics. IEEE J. Solid-State Circuits.

[10] Li J., Du M. Fuzzy Modeling and Compensation of Scale Factor for MEMS Gyroscope.

[11] Zhou H., Hu H. (2010). Reducing drifts in the inertial measurements of wrist and elbow positions. IEEE Trans. Instrum. Meas.

[12] Olivares A., Górriz J.M., Ramírez J. (2011). Accurate human limb angle measurement: sensor fusion through Kalman, least mean squares and recursive least-squares adaptive filtering. Meas. Sci. Technol.

[13] Ashutosh S., Gupta G., Gerasimov V., Ourselin S. In Use Parameter Estimation of Inertial Sensors by Detecting Multilevel Quasi-static States.

[14] Godha S., Lachapelle G., Cannon M.E. Integrated GPS/INS System for Pedestrian Navigation in a Signal Degraded Environment.

[15] Kwakkel S.P., Lachapelle G., Cannon M.E. GNSS Aided *In Situd* Human Lower Limb Kinematics During Running.

[16] Torres-Solis J., Chau T. (2010). Wearable indoor pedestrian dead reckoning system. Pervasive Mob. Comput.

[17] Veltink P.H., Bussmann HansB. J., de Vries W., Martens W.J., van Lummel R.C. (1996). Detection of static and dynamic activities using uniaxial accelerometers. IEEE Trans. Rehabil. Eng.

[18] Krach B., Robertson P. Integration of Foot-Mounted Inertial Sensors into a Bayesian Location Estimation Framework.

[19] Ojeda L., Borenstein J. (2007). Non-GPS navigation for security personnel and first responders. J. Navig.

[20] Skog I., Handel P., Nilsson J.O., Rantakokko K. (2010). Zero-velocity detection—An algorithm evaluation. IEEE Trans. Biomed. Eng.

[21] Ramírez J., Segura J.C., Benítez C., de la Torre A., Rubio A. (2004). Efficient voice activity detection algorithms using long-term speech information. Speech Commun.

[22] Ramírez J., Górriz J.M., Segura J.C., Puntonet C.G., Rubio A. (2006). Speech/non-speech discrimination based on contextual information integrated bispectrum LRT. IEEE Signal Process. Lett.

[23] Górriz J.M., Ramírez J., Puntonet C.G., Segura J.C. (2006). Generalized LRT-based voice activity detector. IEEE Signal Process. Lett.

[24] Ramírez J., Segura J.C., Górriz J.M., García L. (2007). Improved voice activity detection using contextual multiple hypothesis testing for robust speech recognition. IEEE Trans. Speech Audio Process.

[25] Nikovski D., Jain A. Memory-Based Algorithms for Abrupt Change Detection in Sensor Data Streams.

[26] Page E.S. (1954). Continuous inspection schemes. Biometrika.

[27] Basseville M., Nikiforov I.V. (1993). Detection of Abrupt Changes: Theory and Application.

[28] Hastie T., Tibshirani R., Friedman J.H. (2001). The Elements of Statistical Learning.

[29] Olivares A., Olivares G., Mula F., Górriz J.M., Ramírez J. (2011). Wagyromag: Wireless sensor network for monitoring and processing human body movement in healthcare applications. J. Syst. Archit.

[30] Analog Devices (2009). ADXL335 Datasheet. http://www.analog.com/static/imported-files/data_sheets/ADXL335.pdf.

[31] ST Microelectronics (2009). Pitch and Roll LPR550AL Gyroscope. http://www.st.com/stonline/products/families/sensors/datasheets/lpr550al.pdf.

[32] ST Microelectronics (2009). Yaw LY550ALH Gyroscope. http://www.st.com/stonline/products/literature/ds/15802/ly550alh.pdf.

[33] Honeywell (2010). 3-Axis HMC5843 Digital Compasss. http://www.honeywell.com/sites/servlet/com.merx.npoint.servlets.DocumentServlet?docid=DA9ACFE3C-F7C0-9998-6085-D9D84941499D.

[34] Microchip (2009). MCP9700A Analog Temperature Sensor. http://ww1.microchip.com/downloads/en/DeviceDoc/21942e.pdf.

